# Supplementary material for: International Clones of High Risk of Acinetobacter Baumannii—Definitions, History, Properties and Perspectives
Source: Microorganisms. 2023 Aug 19;11(8):2115. doi: 10.3390/microorganisms11082115 (PMC10459012; doi:10.3390/microorganisms11082115)
Supplement: Supplementary file 1 [file microorganisms-11-02115-s001.zip › figures S1 and S2.pdf]

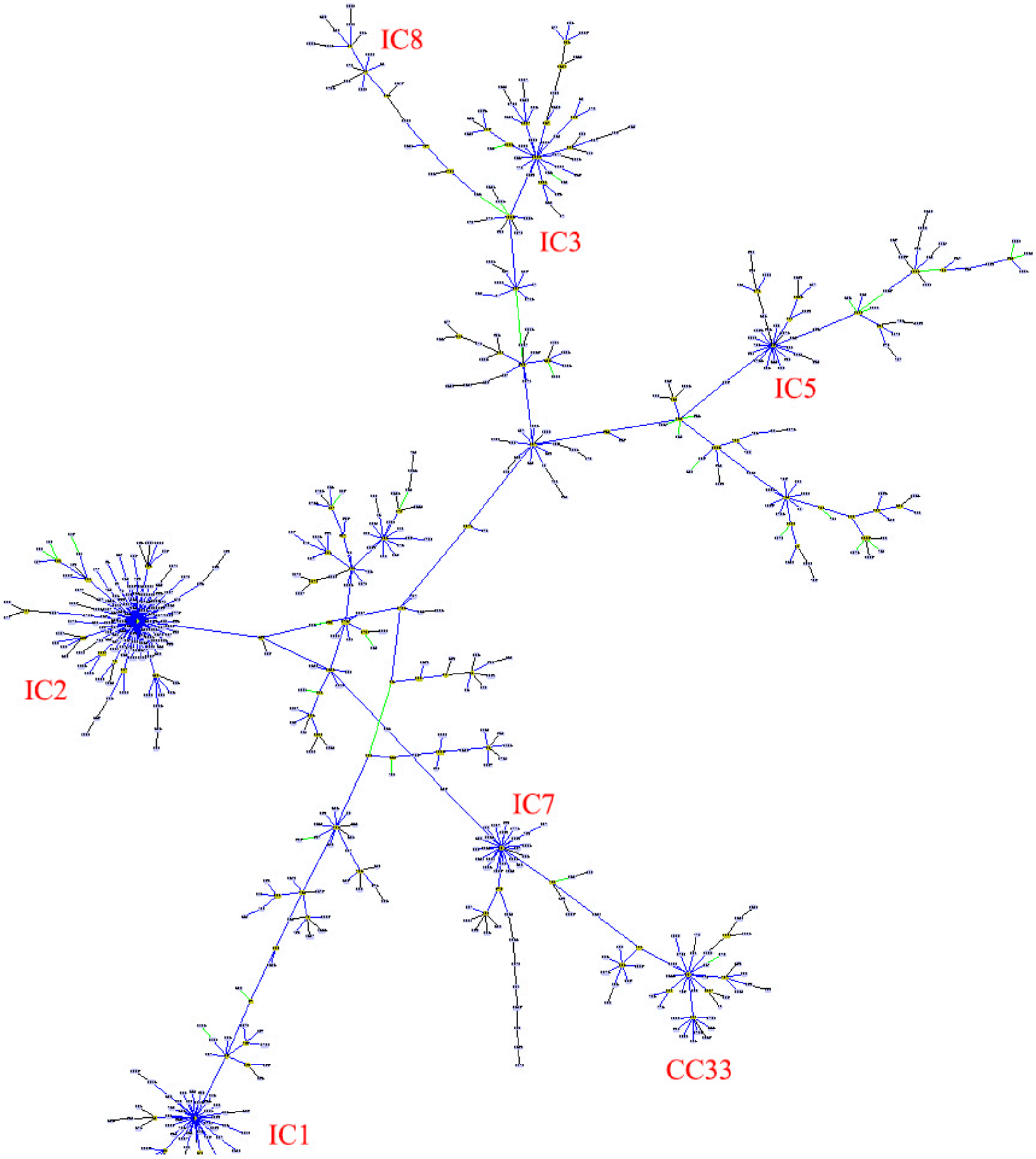

Figure S1. Complete eBURST results for grouping Pasteur STs of *A. baumannii*.

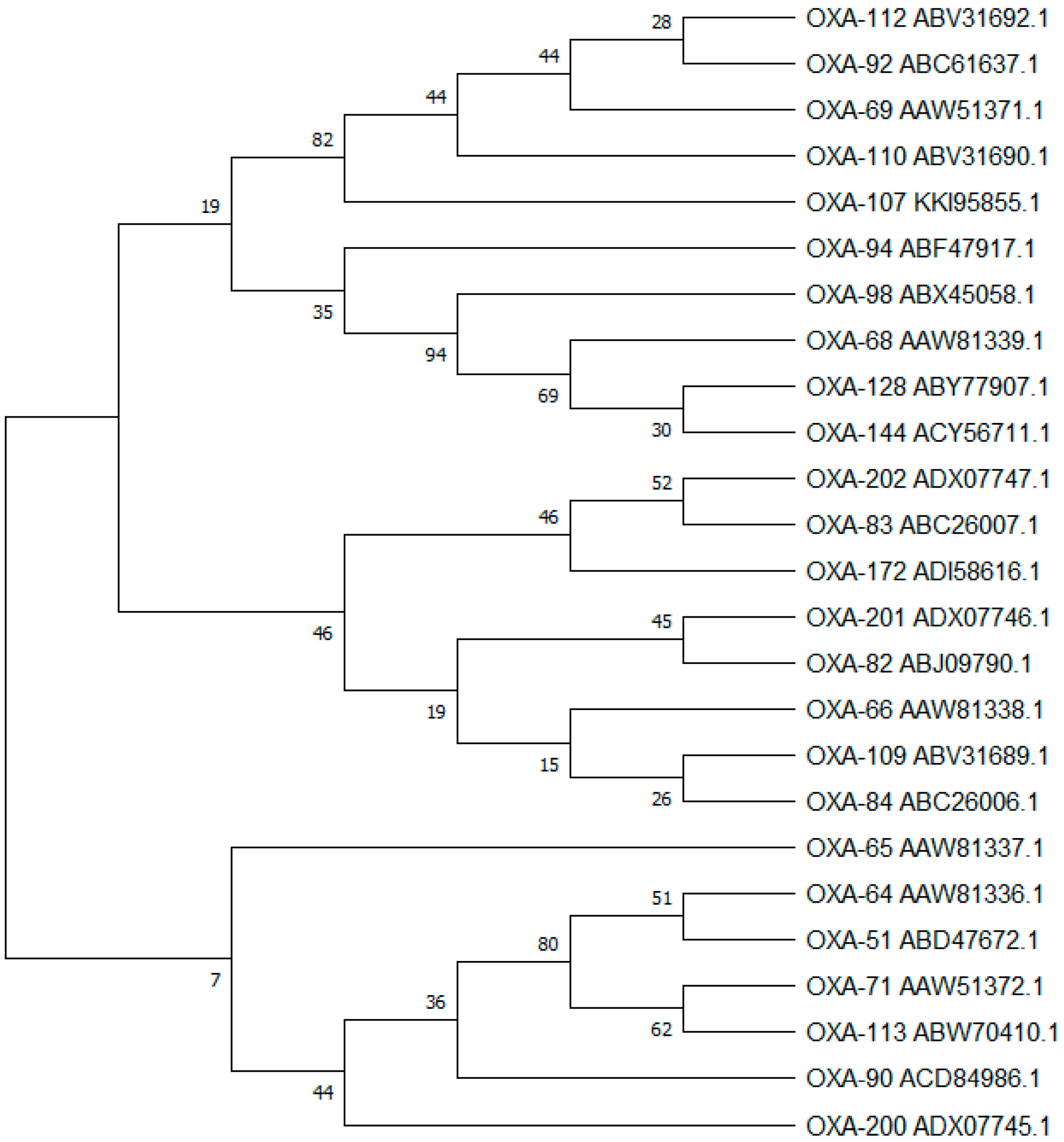

Figure S2. Maximum likelihood tree for amino acid sequences of OXA-51 variants corresponding to IC1-IC9.
